# COVID-19–Related Rumor Content, Transmission, and Clarification Strategies in China: Descriptive Study

**DOI:** 10.2196/27339

**Published:** 2021-12-23

**Authors:** Peishan Ning, Peixia Cheng, Jie Li, Ming Zheng, David C Schwebel, Yang Yang, Peng Lu, Li Mengdi, Zhuo Zhang, Guoqing Hu

**Affiliations:** 1 Department of Epidemiology and Health Statistics Hunan Provincial Key Laboratory of Clinical Epidemiology Xiangya School of Public Health, Central South University Changsha China; 2 Department of Psychology University of Alabama at Birmingham Birmingham, AL United States; 3 Department of Biostatistics College of Public Health and Health Professions University of Florida Gainesville, FL United States; 4 Department of Sociology Central South University Changsha China; 5 National Clinical Research Center for Geriatric Disorders Xiangya Hospital Central South University Changsha China

**Keywords:** COVID-19, rumor, strategy, China, social media

## Abstract

**Background:**

Given the permeation of social media throughout society, rumors spread faster than ever before, which significantly complicates government responses to public health emergencies such as the COVID-19 pandemic.

**Objective:**

We aimed to examine the characteristics and propagation of rumors during the early months of the COVID-19 pandemic in China and evaluated the effectiveness of health authorities’ release of correction announcements.

**Methods:**

We retrieved rumors widely circulating on social media in China during the early stages of the COVID-19 pandemic and assessed the effectiveness of official government clarifications and popular science articles refuting those rumors.

**Results:**

We show that the number of rumors related to the COVID-19 pandemic fluctuated widely in China between December 1, 2019 and April 15, 2020. Rumors mainly occurred in 3 provinces: Hubei, Zhejiang, and Guangxi. Personal social media accounts constituted the major source of media reports of the 4 most widely distributed rumors (the novel coronavirus can be prevented with “Shuanghuanglian”: 7648/10,664, 71.7%; the novel coronavirus is the SARS coronavirus: 14,696/15,902, 92.4%; medical supplies intended for assisting Hubei were detained by the local government: 3911/3943, 99.2%; asymptomatically infected persons were regarded as diagnosed COVID-19 patients with symptoms in official counts: 322/323, 99.7%). The number of rumors circulating was positively associated with the severity of the COVID-19 epidemic (ρ=0.88, 95% CI 0.81-0.93). The release of correction articles was associated with a substantial decrease in the proportion of rumor reports compared to accurate reports. The proportions of negative sentiments appearing among comments by citizens in response to media articles disseminating rumors and disseminating correct information differ insignificantly (both correct reports: χ_1_^2^=0.315, *P*=.58; both rumors: χ_1_^2^=0.025, *P*=.88; first rumor and last correct report: χ_1_^2^=1.287, *P*=.26; first correct report and last rumor: χ_1_^2^=0.033, *P*=.86).

**Conclusions:**

Our results highlight the importance and urgency of monitoring and correcting false or misleading reports on websites and personal social media accounts. The circulation of rumors can influence public health, and government bodies should establish guidelines to monitor and mitigate the negative impact of such rumors.

## Introduction

Accurate, timely, and publicly accessible information is critical to effectively control serious public health emergencies such as the COVID-19 pandemic [1]. Unfortunately, necessary information is sometimes unavailable to the public, and some news reports are misleading or inaccurate. The absence of proper information offers fertile ground for the emergence and propagation of rumors (also called *fake news*). If rumors appear convincing and are not effectively refuted, they can create serious consequences such as poor health-related decisions and distrust of public health agencies by the public.

Over the past few decades, rumor-debunking strategies such as fact checking, corrections, and retractions (eg, deleting social media posts) have been implemented as postevent responses to counteract the impact of rumors [2-4]. The effectiveness of these strategies has not been assessed systematically; previous research focuses on strategies to mitigate public belief of the rumors [5-7] but not on the role of strategies in reducing the proportion of rumor reports and alleviating negative sentiments among the public. Partly due to lack of solid research evidence, the World Health Organization (WHO) concluded that health-related rumors were poorly managed in nearly all major public health emergency events during the 21st century [1].

During the COVID-19 pandemic, rumors have emerged and propagated in nearly every country [8]. The rapid development and widespread use of the internet, social media, and mobile phone technology facilitate the emergence and propagation of rumors, making prevention and control of rumors more challenging than they were even a decade ago. In fact, widespread fake news rumors, such as “COVID-19 is just a version of seasonal influenza” and “Hydroxychloroquine is an effective drug to treat COVID-19,” significantly compromised COVID-19 prevention and control efforts and influenced social stability in highly populated countries such as the United States and Brazil [9].

China began facing large waves of pandemic-related rumors early in 2020. Between February and May 2020, the Chinese government actively released multiple clarification and correction announcements to reduce the influence of those rumors [10]. No empirical research has examined the characteristics of the COVID-19–related rumors or the effectiveness of the counteractive measures taken by Chinese health authorities. Knowledge of these characteristics would be helpful, both for countries across the globe that continue to face COVID-19–related rumors and public health challenges as well as to handle rumor patterns that emerge from future public health emergencies.

This study was designed to examine the characteristics and propagation of rumors during the early months of the COVID-19 pandemic in China and to evaluate the effectiveness of health authorities’ correction announcements. We considered the following research questions: What were the contents of rumors about COVID-19 in the early months of the pandemic in China, and what was the source of those rumors? Was the release of correction announcements by health authorities effective in mitigating the impact of major rumors?

## Methods

### Classification of the COVID-19 Rumors

Rumors were defined as reports that disseminate false information—information that is inconsistent with existing scientific evidence. In this study, all COVID-19 rumors were confirmed to be incorrect based on current scientific evidence by health authorities. In 3 rounds of discussions, we categorized the rumors that we identified into 8 groups: (1) prevention, (2) diagnosis/treatment/assistance, (3) origin and spread of COVID-19, (4) consequences of COVID-19, (5) disease statistics, (6) return to work or back to school, (7) imported cases, and (8) all others. We arranged a trained researcher to categorize each rumor into 1 of the 8 categories. One-third of the rumors were randomly extracted for a second evaluation by 3 other independent researchers. Consistency between both sets of evaluations was excellent (κ=0.96, *P*<.001).

### Data Sources

Data sources were derived through 4 steps. First, based on preliminary search results, we identified 20 prominent and publicly accessible web-based platforms available in China to search for COVID-19–related rumors. These platforms included 9 websites, 5 official Sina Microblog accounts, and 6 WeChat public accounts (Table S1 in Multimedia Appendix 1).

Second, we retrieved all media reports regarding 4 major rumors and classified the media reports into 3 categories: those disseminating rumors, those providing correct information, and those with ambiguous information. We conducted this classification using the ZhongQing HuaYun web-based public service platform (CYYUN), a free public database platform licensed by the Central Committee of the Communist Youth League. CYYUN automatically retrieves media reports from WeChat accounts officially certified by governmental or business entities, Microblog accounts, public forums, news websites, print media, blog accounts, videos, news apps, major overseas media sources, and other mainstream national news sources. CYYUN data are updated approximately every 5 minutes.

Third, Sina Microblog Topic was used to gather readers’ comments in response to media reports of the rumor event with the most public comments. Sina Microblog Topic is a webpage that summarizes personal microblogs with a tag (“#topics-related keywords#”), allowing readers to post personal comments below the microblogs. Comment data from Sina Microblog Topic are freely accessible in China.

Last, the number of daily confirmed COVID-19 cases at the national and provincial levels were derived from the official website of the National Health Commission of China.

### Data Collection

#### COVID-19 Rumor Reports

Given the pattern of the COVID-19 epidemic period in China, we limited the study time period from December 1, 2019 to April 15, 2020. We used Python (version 3.7) to develop a web crawler algorithm to automatically retrieve all media reports related to COVID-19 rumors from the 20 selected web-based platforms. Duplicate and irrelevant reports were removed by manually reviewing article titles and full-text articles.

#### Stages of the Early COVID-19 Epidemic in China

As defined by a previous study [11], the early phase of the COVID-19 pandemic in China was divided into 6 stages: (1) an early stage without any significant interventions (December 30, 2019-January 9, 2020); (2) massive population migration before the Spring Festival but no strong interventions were implemented (January 10, 2020-January 22, 2020); (3) city lockdowns, traffic suspension, and home quarantine (January 23, 2020-February 1, 2020); (4) centralized quarantine and treatment in designated hospitals or facilities, with improved medical resources (February 2, 2020-February 16, 2020); (5) centralized quarantine and whole-community symptom survey administered concerning COVID-19 symptoms, such as fever and respiratory symptoms (February 17, 2020-March 10, 2020); and (6) a focus on preventing imported cases (March 11, 2020-April 15, 2020).

#### Media Reports Related to Major COVID-19 Rumors

The number of relevant media reports obtained from CYYUN was used to estimate the social influence of rumors. We selected 1 rumor that was most socially influential from each of the 6 epidemic stages based on the number of relevant media reports. We retained 4 rumors for analysis; we excluded 2 rumors that attributed false quotes to Professor Nanshan Zhong, a famous Chinese scientist. The rationale of this exclusion is that the high social status of Professor Zhong rather than (or along with) the contents of these quotes might have fueled the spread of the 2 rumors, potentially leading to bias in our analysis of the impact of the contents of rumors.

We used CYYUN to collect all media reports related to the propagation of the 4 retained rumors: Case A, the novel coronavirus can be prevented with “Shuanghuanglian,” a traditional Chinese medicine; Case B, the novel coronavirus is the SARS coronavirus; Case C, medical supplies intended for assisting Hubei were detained by the local government; and Case D, asymptomatically infected persons were regarded as diagnosed COVID-19 patients with symptoms in official counts. (Note that Case D is a rumor because the WHO and all major countries do not count asymptomatically infected persons as diagnosed COVID-19 cases in their official data counts [12]).

All media reports related to the 4 major rumors were gathered and processed. Based on official statements from the government regarding each rumor, trained researchers manually divided each media report into 3 categories: (1) the report disseminated or perpetuated the rumor, (2) the report disseminated correct information to refute the rumor, or (3) the report disseminated ambiguous information that did not clearly support or refute the rumor. To assure the consistency of the classifications, we randomly selected 500 media reports for each of the 4 major rumors and asked an independent researcher to categorize them into the 3 categories. Consistency between both sets of researchers was acceptable (Case A: κ=0.87, *P*<.001; Case B: κ=0.87, *P*<.001; Case C: κ=0.90, *P*<.001; Case D: κ=0.87, *P*<.001).

#### Microblog Readers’ Comments

We used a Python crawler algorithm to retrieve reader comments concerning Microblog articles for the most discussed rumor (Case A) on Sina Microblog Topic, “The novel coronavirus can be prevented with “Shuanghuanglian.” We selected Case A for this analysis because it was sustained for a longer time period than the other rumors. It also stimulated more media reports and readers’ comments compared to the other 3 cases. Using the sentiment orientation analysis application programming interface from Baidu [13], we grouped reader comments into positive (expressing agreement, support, or optimistic attitudes, eg, “the situation will surely become better”); neutral (showing neither clear support and optimism, nor disagreement and pessimism, eg, “thanks for interacting with us”); or negative (expressing disagreement or worried, pessimistic, and ironic attitudes, eg, “I feel like swearing”) categories.

### Data Analysis

We implemented 2 strategies to assess the effectiveness of authorities’ refutation of rumors.

We compared the distribution of the 2 types of media reports (those perpetuating the rumor and those correcting the rumor) before and after the rumor was formally refuted in the media. Because there is no standard criterion to determine the launch time of a formal rumor-refuting effort, we studied the temporal distributions of the number of correct reports and the number of rumor reports per hour for all 4 cases (Figure S1 in Multimedia Appendix 1). Based on these empirical distributions, we found that the number correct reports per hour generally increased substantially in subsequent hours once it reached over 50 reports. Thus, we considered the refutation as formally launched from the first hour in which more than 50 reports refuting the rumor were posted. We considered 3 phases for our analysis: prerefutation, refuting the rumor, and postrefutation.

We examined differences in the distribution of positive, neutral, or negative emotions in comments responding to reports disseminating the rumor and in reports correcting the rumor. We focused this analysis on case A, the rumor that received the most reader comments and was sustained the longest, thus having the largest social impact among all COVID-19 rumors during the early stages of the pandemic in China. To conduct the analysis, we examined both the frequency of reader comments and the time intervals between the first and last comments from the same readers when more than 2 comments were posted.

Based on publicly accessible Microblog users’ unique identity accounts, we compared the distribution of comments in response to case A from readers who posted 2 comments or more. For each user, the first and last comments during the study period were considered, and the pairs were divided into 4 groups: Group A, both comments occurred in response to reports disseminating correct information; Group B, both comments occurred in response to reports disseminating rumors; Group C, the first comment occurred in response to a report disseminating a rumor, and the last in response to correct information; and Group D, the first comment occurred in response to a report disseminating correct information and the last in response to a rumor. This grouping allowed us to examine the potential effect of the order of reading media reports (first reading reports disseminating rumors and then reading correct reports or vice versa).

All statistical analyses were performed using the R software package (version 4.0.0). Proportions and Spearman rank correlation coefficient with 95% confidence intervals were calculated. The chi-square test was used to test the difference in the proportion of rumor reports and correct reports between prerefutation and postrefutation. All tests were 2 tailed, and *P*<.05 was considered statistically significant.

### Ethics Concerns

This study was approved by the ethics committee of Xiangya School of Public Health (XYGW-2020-43). This study used open-access social media data and excluded all personal information. The ethics committee determined that this study was exempt from requiring informed consent.

## Results

### Characteristic of Rumors

After screening 19,683 recorded rumors on 20 websites and social media accounts, we obtained 1829 unique COVID-19–related rumors in China that appeared from December 1, 2019 to April 15, 2020 (Figure S2 in Multimedia Appendix 1).

**Figure 1:**
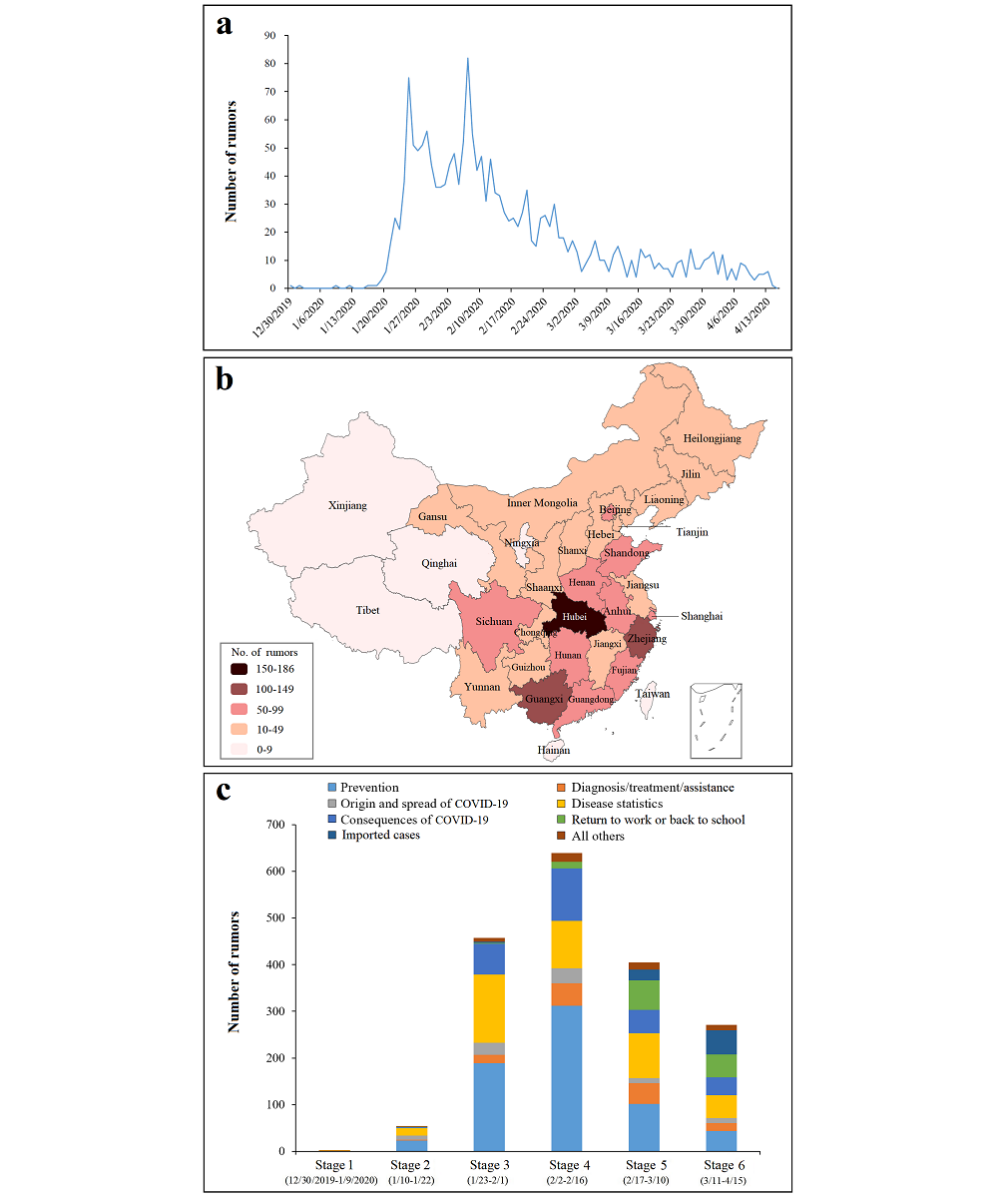
Number of rumors related to the COVID-19 epidemic in China from December 30, 2019 to April 15, 2020 (a. time trend; b. provincial variations; c. contents by stage).

The frequency of rumors began with a quiescent period (<6 rumors per day before January 20, 2020), but then rose rapidly to reach an initial peak on January 25, 2020 (n=75). After a second peak on February 7, 2020 (n=82), the frequency started to decrease gradually (Figure 1a). “Pneumonia,” “WeChat,” and “spread” were the most commonly occurring words, with high-frequency words varying across the 6 stages of the COVID-19 epidemic (Table S2 and Figure S3 in Multimedia Appendix 1).

Figure 1b shows the large variation in the number of rumors across different provinces during the study period. Of the 1829 rumors, 399 (21.8%) rumors involved more than 1 province, but the majority (1430 rumors) involved a single province. The largest number occurred in Hubei (n=186), followed by Zhejiang (n=137) and Guangxi (n=121). Ningxia, Tibet, Xinjiang, Qinghai, Hainan, and Taiwan each had fewer than 10 rumors.

The content of the rumors evolved greatly across the 6 stages of the COVID-19 epidemic in China (Figure 1c). Rumors related to prevention and disease statistics were most common, accounting for 72.2% (39/54), 73.3% (335/457), and 64.8% (414/639) of all rumors in stages 2, 3, and 4, respectively. The percentage of rumors involving the categories *diagnosis/treatment/assistance* (χ_1_^2^=6.352, *P*=.01), *return to work or back to school* (χ_1_^2^=148.094, *P*<.001), and *imported cases* (χ_1_^2^=126.04, *P*<.001) significantly increased during stages 5 and 6.

**Figure 2:**
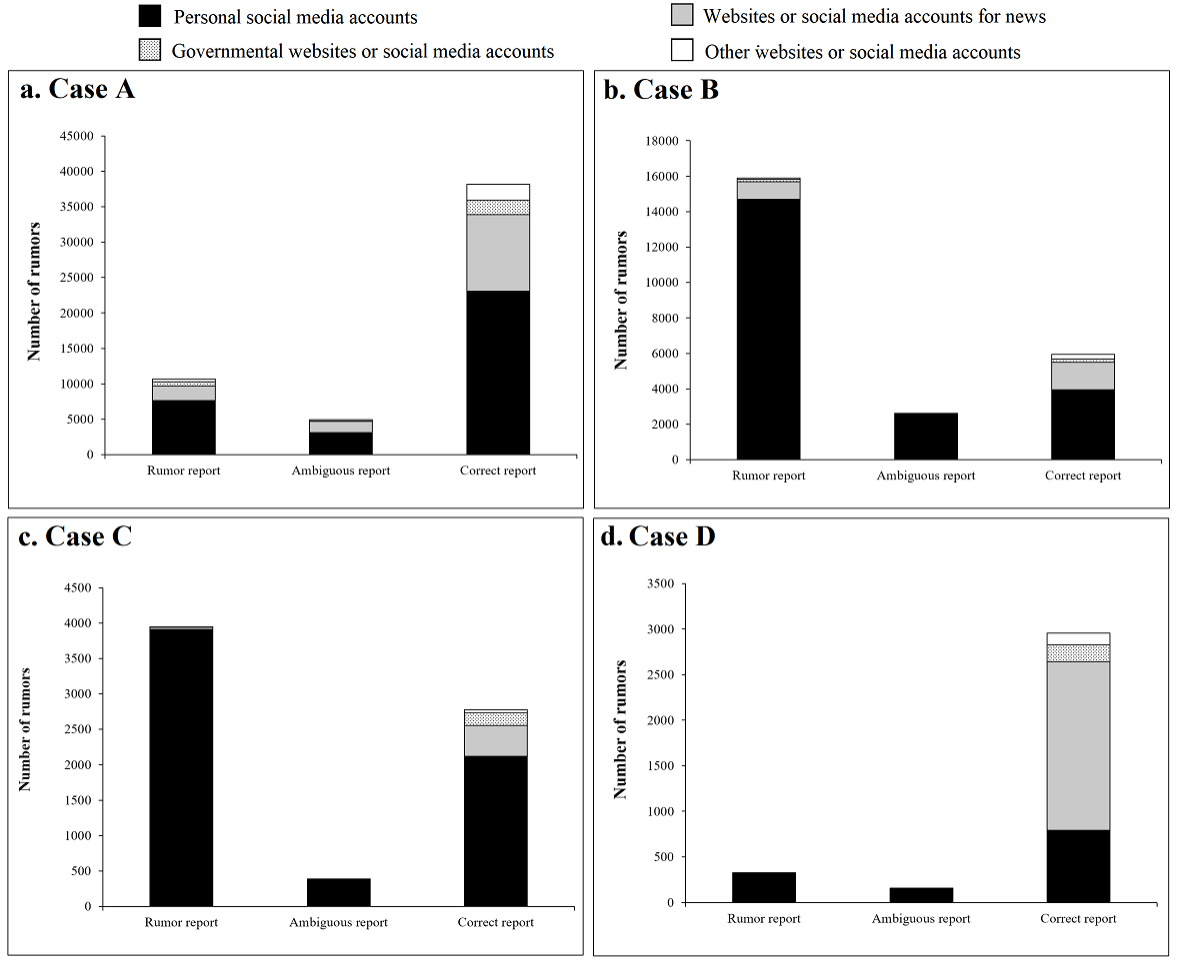
Source of media reports for 4 major rumors related to the COVID-19 epidemic in China.

We considered in greater detail the 4 most widespread rumor cases (case A, the novel coronavirus can be prevented with “Shuanghuanglian”; case B, the novel coronavirus is the SARS coronavirus; case C, medical supplies intended for assisting Hubei were detained by the local government; and case D, asymptomatically infected persons were regarded as diagnosed COVID-19 patients with symptoms in official counts). Most reports were from personal social media accounts for case A (33,870/53,798, 63.0%), case B (21,234/24,436, 86.9%), and case C (6411/7101, 90.3%). For case D, most reports were from websites and social media accounts of news agencies (1850/3436, 53.8%) (Figure 2). Personal social media accounts were the major source of rumor reports (case A: 7648/10,664, 71.7%; case B: 14,696/15,902, 92.4%; case C: 3911/3943, 99.2%; case D: 322/323, 99.7%) and ambiguous reports (case A: 3116/4968, 62.7%; case B: 2582/2592, 99.6%; case C: 382/383, 99.7%; case D: 156/156, 100.0%).

In addition, there was a strong correlation between the frequency of rumors and the daily number of newly confirmed COVID-19 cases at the national level from December 30, 2019 to April 15, 2020 (ρ=0.88, 95% CI 0.81-0.93, *P*<.001). A spatial correlation between the frequency of rumors and the cumulative number of confirmed cases up to April 15, 2020 also emerged at the provincial level (ρ=0.83, 95% CI 0.61-0.94, *P*<.001) (Figure S4 in Multimedia Appendix 1).

### Effectiveness of Releasing Correct Information

Totals of 53,798 media reports (case A), 24,436 media reports (case B), 7101 media reports (case C), and 3436 media reports (case D) were obtained after eliminating duplicates (n=3788) and irrelevant reports (n=4036) (Figure S2b in Multimedia Appendix 1).

The proportion of rumor reports significantly decreased after the rumors were refuted by health authorities for case A (before: 534/666, 80.2%; after: 5685/45,657, 12.5%), case C (before: 1978/2101, 94.1%; after: 908/3796, 23.9%), and case D (before: 120/860, 14.0%; after: 192/2431, 7.9%) (*P*<.001) (Table 1); however, the proportion of rumor reports increased from 10.8% (50/463) to 60.5% (12,004/19,846) for case B after the official clarification with correction information was released (*P*<.001).

**Figure 3:**
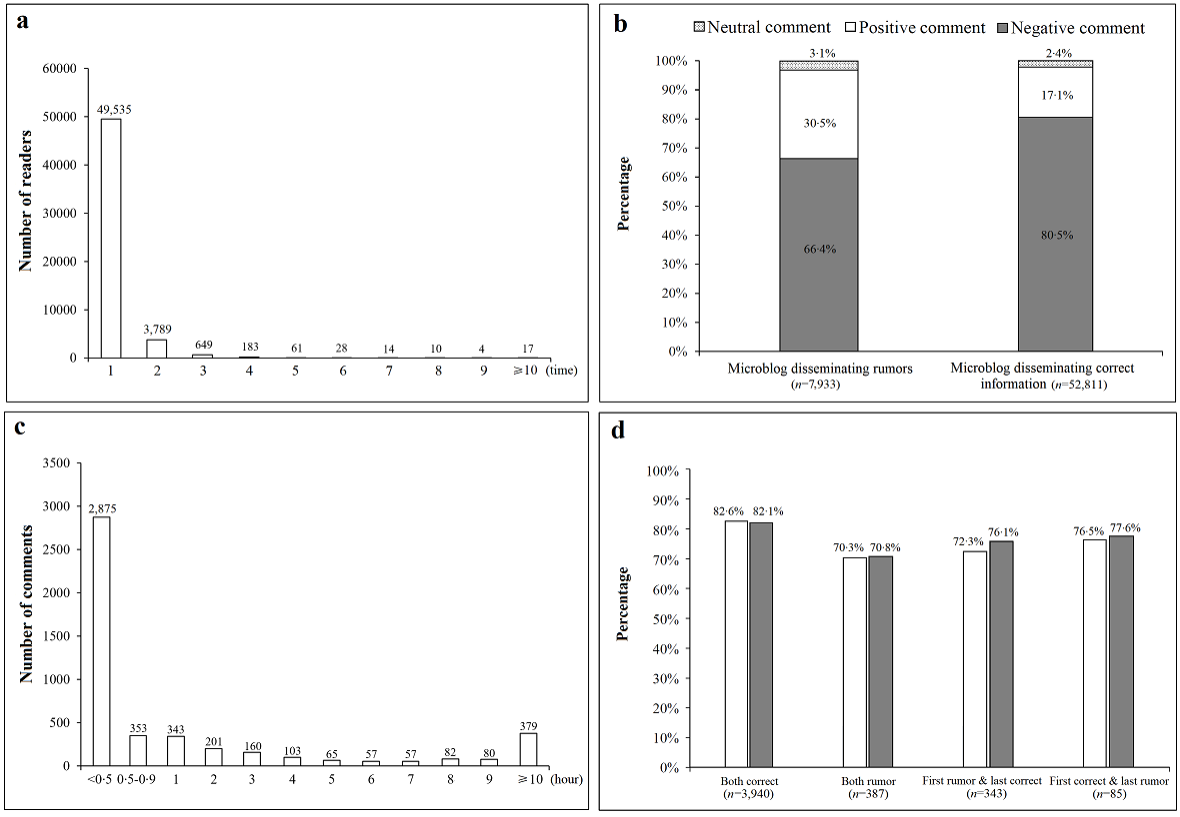
Readers’ comments in response to microblog articles for the case A rumor, “the novel coronavirus can be prevented with ‘Shuanghuanglian’”: (a) frequency of comments, (b) comment distribution by type of article, (c) time interval between the first and last comment for the same readers, and (d) proportion of negative comments in the first and last comments by type of article.

For case A (the novel coronavirus can be prevented with “Shuanghuanglian”), we identified a total of 60,744 comments posted by 54,290 microblog readers in response to 42 articles disseminating rumors and 78 articles refuting rumors and providing accurate information. Most readers posted once (49,535/54,290, 91.2%) or twice (3789/54,290, 7.0%) (Figure 3a), and most readers’ comments expressed emotions with a negative connotation (eg, anger, anxiety, fear) in response to both types of microblog articles. However, the proportion of comments with negative emotions for articles disseminating correct information was significantly higher than the proportion for articles disseminating rumors (correct: 42,485/52,811, 80.5%; rumor: 5271/7933, 66.4%, χ_1_^2^=804.55, *P*<.001) (Figure 3b). Among readers who posted multiple comments, 60.5% (2875/4755) posted their first and last comments within half an hour (Figure 3c), and the proportion of negative comments posted by those readers did not change significantly when comparing their first versus last posts, no matter what type of article they read (both correct: χ_1_^2^=0.315, *P*=.58; both rumors: χ_1_^2^=0.025, *P*=.88; first rumor and last correct: χ_1_^2^=1.287, *P*=.26; first correct and last rumor: χ_1_^2^=0.033, *P*=.86) (Figure 3d).

**Table 1:** Media reports related to 4 most influential rumor cases before and after refuting the rumor.

Case and time period			Rumor report, n (%)		Correct report, n (%)		*P* value
**Case A: The novel coronavirus can be prevented with “Shuanghuanglian”**							<.001
	Prerefutation	534 (80.2)		81 (12.2)			
	Refuting the rumor (January 31, 11 PM to midnight)	4445 (59.5)		2079 (27.8)			
	Postrefutation	5685 (12.5)		36006 (78.9)			
**Case B: The novel coronavirus is the SARS coronavirus**							<.001
	Prerefutation	50 (10.8)		412 (89.0)			
	Refuting the rumor (February 9, 9 PM to 10 PM)	3848 (93.2)		86 (2.1)			
	Postrefutation	12004 (60.5)		5444 (27.4)			
**Case C: Medical supplies intended for assisting Hubei were detained by the local government**							<.001
	Prerefutation	1978 (94.1)		3 (0.1)			
	Ongoing (February 10, 11 PM to midnight)	1057 (87.8)		51 (4.2)			
	Postrefutation	908 (23.9)		2721 (71.7)			
**Case D: Asymptomatically infected persons were regarded as diagnosed COVID-19 patients with symptoms in official counts**							<.001
	Prerefutation	120 (14.0)		660 (76.7)			
	Refuting the rumor (March 22, 7 AM to 8 AM)	11 (7.6)		126 (86.9)			
	Postrefutation	192 (7.9)		2171 (89.3)			

## Discussion

### Principal Findings

This study yielded 3 key findings. First, the contents and the number of rumors were strongly associated with the development of the COVID-19 epidemic in China, both temporally and geographically, and reports from personal social media accounts accounted for 71.7% (7648/10,664), 92.4% (14,696/15,902), 99.2% (3911/3943), and 99.7% (322/323) for cases A, B, C, and D, respectively, of all rumor reports. Second, releasing news to correct rumors decreased the dissemination of media articles for 3 of the 4 selected rumors (cases A, C, and D) but did not reduce news disseminations for case B. Third, the likelihood that readers posted negative comments in response to rumor reports and to reports disseminating correct information differed insignificantly (both correct reports: χ_1_^2^=0.315, *P*=.58; both rumors: χ_1_^2^=0.025, *P*=.88; first rumor and last correct report: χ_1_^2^=1.287, *P*=.26; first correct report and last rumor: χ_1_^2^=0.033, *P*=.86).

### Interpretation of Findings

The findings of previous studies, regarding fluctuations in the number of rumors reported on a daily basis, the geographic variations for those reports across China [14], and major topics of the COVID-19 rumors [14,15], were generally concordant with our findings. The strong and positive correlation between reported rumors and the development of the COVID-19 epidemic likely reflects extreme tension, fear, and anxiety about the novel contagious disease among the population, especially residents of the regions most affected by the epidemic [16], such as Hubei, Zhejiang, and Guangxi. The likely reason for this was that the rapidly growing epidemic provoked emotional responses among inhabitants, leading them to scramble for any information they could find to alleviate their anxieties and fears. When the residents in those regions were unable to obtain timely official answers about major public concerns related to the COVID-19 epidemic, rumors emerged and were disseminated quickly [17]. For example, during the early stages of the COVID-19 epidemic in China, the question of how to dispose of used face masks emerged as a major public health concern. The question aroused 2031 microblog posts, but only 10 official Sina Microblog accounts released authoritative information on the issue before January 23, 2020 [18]. As a result, a total of 14 versions of misleading rumors emerged immediately on this topic, and 4740 articles disseminated improper strategies to dispose of discarded masks, such as heating them in the oven, scrubbing them with water, or cutting them into pieces [18].

This study presents a unique finding that personal social media accounts were the dominant source of rumor reports. This may reflect the combined effect of flourishing personal social media accounts [19], an inability for average citizens to detect misinformation [20], and amplification effects through (ie, an “echo chamber”—people are susceptible to peer influences when engaging in a homogeneous online cluster with others who share similar interests) [21].

Unexpectedly, we found that websites or news-based social media accounts were the second most common source of rumor reports. This may be a result of inadequate fact-checking of contents by the news industry or deliberate reports designed to attract public attention to these news media outlets [22,23]. As an example, the most widely spread rumor (Case A, the novel coronavirus can be prevented with “Shuanghuanglian,” a traditional Chinese medicine used to treat the common cold) was first released by Weibo of Xinhua, an official news platform with nearly 100 million subscribers [24]. That release quickly led to a Sina Microblog topic titled “The novel coronavirus can be prevented with Shuanghuanglian,” attracting 1.55 billion readings and 423,000 reader comments [25] and leading to panic buying of Shuanghuanglian.

The release of news to correct rumors was successful in reducing the spread of rumors in cases A, C, and D. This confirms that it is possible to mitigate the spread of rumors through authoritative releases of correct information in a timely manner [26]. Case B did not follow the same pattern, probably due to an unintentional slip of tongue by a government spokesperson at the 19th press conference in Hubei province on February 9, 2020 [27]. The spokesperson unintentionally and incorrectly confirmed the rumor, leading to a rapid increase in the number of rumor reports concerning Case B (from 41 to 15,767; Figure S5 in Multimedia Appendix 1) within 3 hours of the start of the press conference.

Strikingly, most reader comments in response to articles were negative, whether the articles reported rumors or correct information, and the proportions of negative comments were similar between both types of articles. These results match those of previous reports [28,29] and may reflect the fact that official correction news tends to disseminate correct information but largely overlooks any attempt to provide emotional support or empathy to affected people. Such factual presentation without empathy leads to unproductive dialogue with the emotional public, who release their negative sentiments in writing [29].

### Implications

Our findings have 3 major implications. First, they underscore the importance of releasing scientifically accurate information in response to major public health concerns in a timely manner. Such releases help prevent the spread of rumors. To be effective, they require close cooperation between government officials, scientists, and the media.

In emerging health crises, such as the COVID-19 pandemic, public concerns inevitably arise that cannot always be answered immediately with accurate scientific evidence. In the absence of guidelines to satisfy public angst and curiosity for critical public health information while science progresses, basic guidance might be established by the WHO or academic experts to direct the release of available information through multiple channels, including government websites, news websites, and official and personal social media accounts. Such releases might reduce tension and anxiety among the public.

Second, the official release of correction articles tended to reduce rumor reports in China, suggesting similar effects could be expected in other countries. Success with this strategy would require a real-time web-based monitoring system to detect rumor reports and close cooperation between government, media companies, and public health experts to release authoritative correction articles that refute rumors, with the caveat that such a strategy may not work well in countries where government and public health experts provide conflicting advice, such as in the United States for much of the situation with COVID-19 in 2020 [30].

Last, our findings suggest rigorous public health research is needed to generate solutions to unsolved questions, including how to effectively detect rumor reports as early as possible, which solutions might be best for releasing and disseminating correction articles, and how to effectively alleviate negative sentiments held by the public. In addition, the effectiveness of alternative postrumor approaches such as fact-checking and retractions (eg, deleting posts) should be evaluated.

### Limitations

This study has limitations. First, our data collection approach likely underestimated the number of COVID-19 rumors, by missing some comments in response to Sina Microblog topics, since some were likely quickly detected and deleted by platform providers as part of efforts to control rumors [31]. Second, the lack of detailed data limited our ability to explore why most reader comments in response to media articles that disseminated correct information were negative and how to alleviate such negative sentiments to promote public support on fighting the COVID-19 pandemic. Further research is needed to address this critical public health matter. Third, we could not determine if any rumors we studied were published artificially by robots. As readers generally do not know who posted the reports, the role of robots is unlikely to influence our findings substantially. However, future studies may collect data on readers’ responses to rumors spread by robots versus those spread by humans to quantify potential differences in their social impact and to develop specific interventions to deal with them separately.

### Conclusion

The number of rumors about COVID-19 fluctuated greatly in China during the early months of the pandemic. The frequency of rumors was highly correlated with the magnitude of the epidemic. Rumor-disseminating reports emerged primarily from personal social media accounts, and releasing rumor-correcting news substantially reduced the spread of rumor reports. Reader comments in response to media articles disseminating rumors and to articles disseminating correct information expressed nearly the same percentage of negative emotions.

Individual governments and relevant international organizations such as the WHO should take immediate actions to develop regulations or guidelines to help social media companies and users of social media prepare and release reports properly and efficiently to prevent and control rumors, especially when scientific knowledge is limited during a health crisis such as the COVID-19 pandemic. In the meantime, our results suggest refutation reports were generally successful in reducing spread of rumors, and governments should encourage timely release of correct information to minimize the impact of rumors.

## Appendix

Multimedia Appendix 1 Supplementary materials.

## Abbreviations

CYYUN: ZhongQing HuaYun web-based public service platform
WHO: World Health Organization
