# Supplementary material for: COVID-19–Related Rumor Content, Transmission, and Clarification Strategies in China: Descriptive Study
Source: J Med Internet Res. 2021 Dec 23;23(12):e27339. doi: 10.2196/27339 (PMC8709421; doi:10.2196/27339)
Supplement: Multimedia Appendix 1 [file jmir_v23i12e27339_app1.docx]

**Appendix figures and tables**

1. Table S1. Data sources (website and social media accounts) summarizing rumors related to the COVID-19 pandemic.

2. Table S2. Frequency of attribute words embedded in COVID-19–related rumors across six stages of the pandemic.

3. Figure S1. The temporal distribution of the correct reports and rumor reports for each of the four major rumors.

4. Figure S2. Flow chart of rumor screening.

5. Figure S3. Word cloud of COVID-19–related rumors across six stages of the pandemic in China.

6. Figure S4. Relationship between number of rumors and the confirmed COVID-19 cases in China.

7. Figure S5. Number of rumor reports related to “*Case* *B*. the novel coronavirus is the SARS coronavirus” in hours.

Table S1. Data sources (website and social media accounts) summarizing rumors related to the COVID-19 pandemic.

| Name | Organization | Type of platform | Number of rumors | URL/WeChat account |
| --- | --- | --- | --- | --- |
| Weibopiyao | Weibo official | Microblog account | 2526 | <https://weibo.com/weibopiyao> |
| Chinese Internet rumor clarification platform | Cyberspace Administration of China | Microblog account | 763 | <https://weibo.com/u/6525943125> |
| Kexuepiyao | China Association for Science and Technology | Microblog account | 3936 | <https://weibo.com/u/6507165034?is_hot=1https://weibo.com/u/6507165034> |
| Zhuoyaoji | Non-government company | Microblog account | 529 | <https://weibo.com/u/6590980486> |
| Piyaoge | Non-government company | Microblog account | 1283 | <https://weibo.com/shuhuiyang> |
| Henanzhuoyaoji | Non-government company | Microblog account | 516 | <https://weibo.com/xinxiangmeishipindao> |
| Jiningquanmeitipiyao | Government of Jining | Microblog account | 439 | <https://weibo.com/6095767028/about> |
| Wenzhoupiyao | Wenzhou debunking rumors report website | Microblog account | 865 | <https://weibo.com/wenzhoupiyao> |
| Shandongpiyao | Shandong Internet Illegal Information Reporting Center | Microblog account | 3150 | <https://weibo.com/shandongpiyao> |
| Chinese Internet rumor clarification platform | Xinhua news, Cyberspace Administration of China | Website | 991 | <http://www.piyao.org.cn/2020yqpy/> |
| Yiqingyaoyanfensuiji | The Central Committee of the Communist Youth League | Website | 453 | <http://2019ncov.gsdata.cn/oem/yaoyan/index.php?sign=249490a3d7ad9c7d> |
| Real-time refutation of COVID-19 rumors | Tencent news | Website | 827 | <https://vp.fact.qq.com/home?state=1&ADTAG=xw-1.jz> |
| COCID-19 rumor ranking | DingXiangWang | Website | 244 | <https://ncov.dxy.cn/ncovh5/view/pneumonia_rumors?from=dxy&source=undefined> |
| A summary of rumors from Heilongjiang traffic broadcast | Traffic Broadcasting of Heilongjiang | Website | 187 | <https://mp.weixin.qq.com/s/oCEwZrLwJyQwe2ZAL8Qlsw> |
| Toutiaopiyao | The official account of Toutiao anti-rumor | WeChat public account | 528 | ttpiyao |
| Yaoyanguolvqi | WeChat official | WeChat public account | 308 | wx-yyglq |
| Wenzhoupiyao | Cyberspace Administration of Wenzhou | WeChat public account | 828 | wenzhoupiyao |
| Fujianpiyao | Fujian Daily, Southeast Network | WeChat public account | 865 | fjwlpy |
| Chengduwangluopiyao | Cyberspace Administration of Chengdu, Chengdu Commercial Daily | WeChat public account | 143 | cdpiyao |
| Nanningpiyao | Cyberspace Administration of Nanning, Nanning Internet Association | WeChat public account | 302 | nanningpiyao |

Table S2. Frequency of attribute words embedded in COVID-19–related rumors across six stages of the pandemic.

| Rank | All periods | |  | Stage 2 | |  | Stage 3 | |  | Stage 4 | |  | Stage 5 | |  | Stage 6 | |
| --- | --- | --- | --- | --- | --- | --- | --- | --- | --- | --- | --- | --- | --- | --- | --- | --- | --- |
|  | Word | Frequency |  | Word | Frequency |  | Word | Frequency |  | Word | Frequency |  | Word | Frequency |  | Word | Frequency |
| 1 | pneumonia | 402 |  | coronavirus | 26 |  | novel | 156 |  | spread | 134 |  | pneumonia | 95 |  | spread | 68 |
| 2 | WeChat | 364 |  | novel | 24 |  | coronavirus | 142 |  | WeChat | 122 |  | COVID-19 | 94 |  | COVID-19 | 63 |
| 3 | spread | 341 |  | pneumonia | 19 |  | pneumonia | 132 |  | video | 112 |  | spread | 83 |  | quarantine | 62 |
| 4 | novel | 301 |  | Wuhan | 18 |  | infection | 109 |  | epidemic | 102 |  | WeChat | 79 |  | back to school | 57 |
| 5 | video | 293 |  | prevention | 17 |  | WeChat | 108 |  | disseminate | 99 |  | epidemic | 62 |  | pneumonia | 56 |
| 6 | coronavirus | 292 |  | infection | 11 |  | Wuhan | 103 |  | pneumonia | 99 |  | video | 62 |  | confirmed | 52 |
| 7 | infection | 273 |  | patient | 10 |  | netizen | 90 |  | infection | 90 |  | confirmed | 61 |  | WeChat | 50 |
| 8 | epidemic | 256 |  | virus | 9 |  | video | 76 |  | novel | 87 |  | quarantine | 53 |  | network transmission | 39 |
| 9 | COVID-19 | 245 |  | netizen | 9 |  | epidemic | 61 |  | moments | 86 |  | back to school | 53 |  | video | 38 |
| 10 | disseminate | 242 |  | spread | 8 |  | WeChat group | 60 |  | netizen | 83 |  | disseminate | 47 |  | disseminate | 34 |
| 11 | netizen | 238 |  | infector | 8 |  | disseminate | 58 |  | mask | 83 |  | infection | 43 |  | case | 34 |
| 12 | quarantine | 234 |  | case | 7 |  | moments | 57 |  | coronavirus | 82 |  | face mask | 42 |  | network | 33 |
| 13 | confirmed | 230 |  | disease | 6 |  | confirmed | 55 |  | disinfection | 78 |  | netizen | 41 |  | moments | 32 |
| 14 | Wuhan | 223 |  | video | 5 |  | repost | 52 |  | virus | 77 |  | WeChat group | 37 |  | virus | 29 |
| 15 | moments | 212 |  | hospital | 5 |  | quarantine | 51 |  | WeChat group | 72 |  | case | 37 |  | epidemic | 27 |

Notes:

a. A word cloud was not calculated in the first stage due to the small number of rumors.

b. The six stages were defined according to the development of the COVID-19 pandemic in China: (1) an early stage without any significant interventions (December 30, 2019-January 9, 2020); (2) massive population migration before the Spring Festival but no strong interventions were implemented (January 10, 2020-January 22, 2020); (3) city lockdown, traffic suspension, and home quarantine (January 23, 2020-February 1, 2020); (4) centralized quarantine and treatment in designated hospitals or facilities, with improved medical resources (February 2, 2020-February 16, 2020); (5) centralized quarantine and whole-community symptom survey administered concerning COVID-19 symptoms, such as fever and respiratory symptom (February 17, 2020-March 10, 2020); and (6) a focus on preventing imported cases (March 11, 2020-April 15, 2020).

**
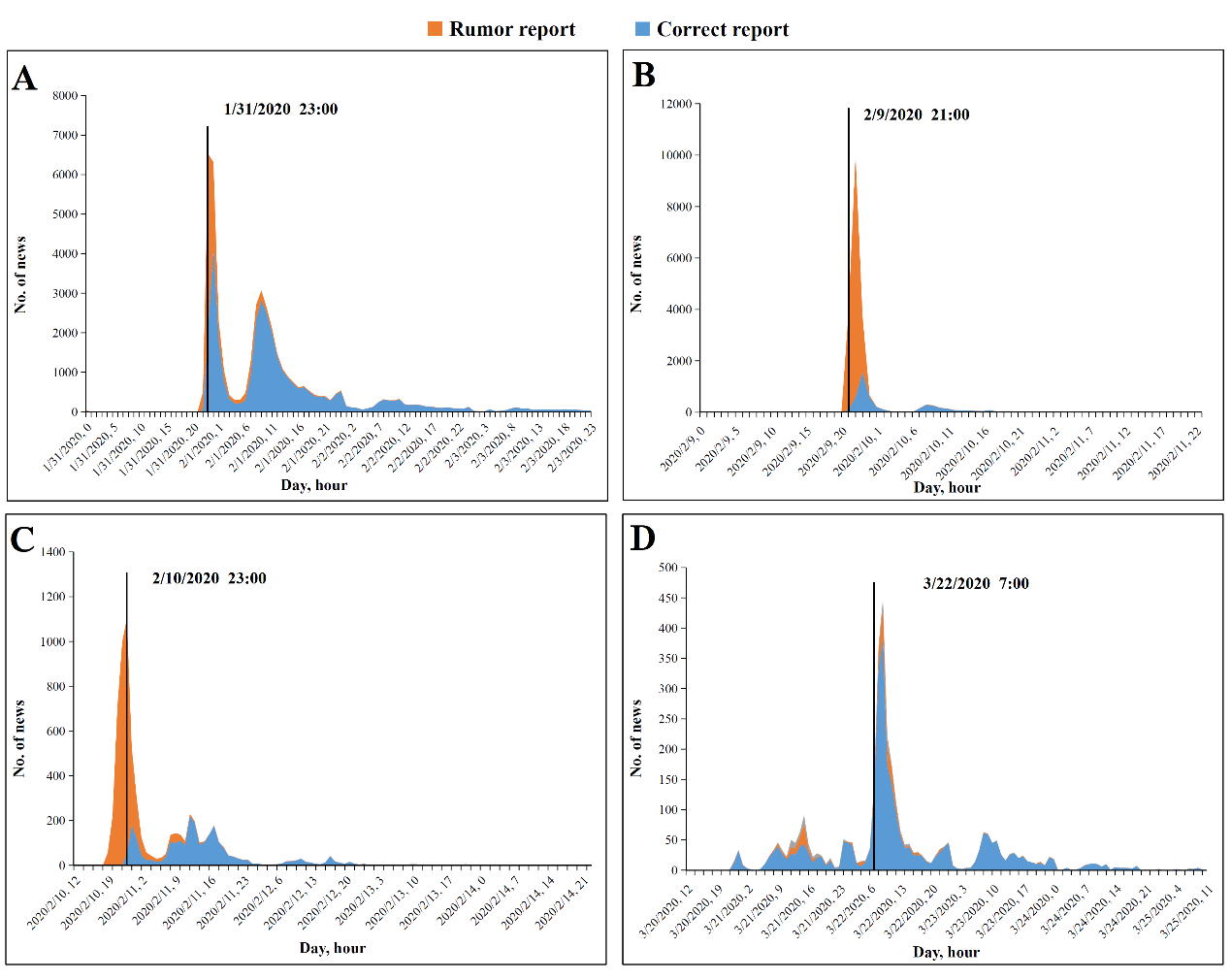
**

Figure S1. The temporal distribution of the correct reports and rumor reports for each of the four major rumors.

**
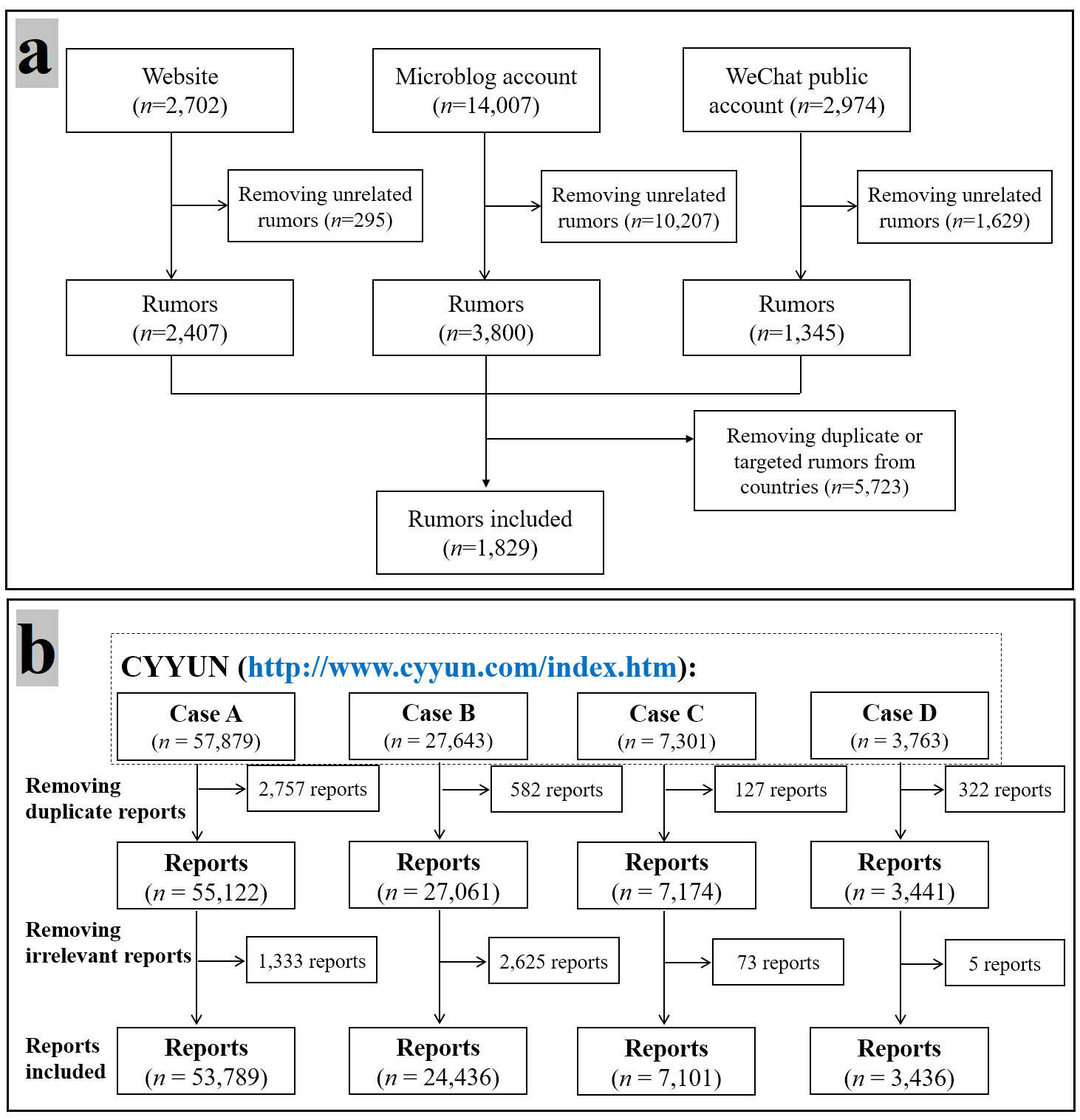
**

Figure S2. Flow chart of rumor screening.


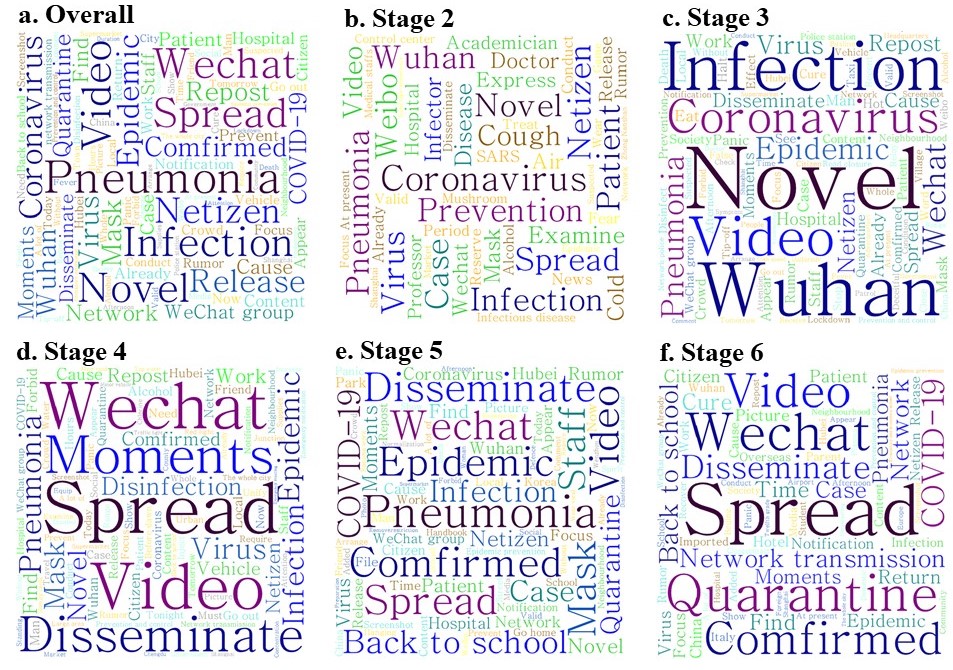


Figure S3. Word cloud of COVID-19–related rumors across six stages of the pandemic in China.

Note:

a. A word cloud was not calculated in the first stage due to the small number of rumors.

b. The six stages were defined according to the development of the COVID-19 pandemic in China: (1) an early stage without any significant interventions (December 30, 2019-January 9, 2020); (2) massive population migration before the Spring Festival but no strong interventions were implemented (January 10, 2020-January 22, 2020); (3) city lockdown, traffic suspension, and home quarantine (January 23, 2020-February 1, 2020); (4) centralized quarantine and treatment in designated hospitals or facilities, with improved medical resources (February 2, 2020-February 16, 2020); (5) centralized quarantine and whole-community symptom survey administered concerning COVID-19 symptoms, such as fever and respiratory symptom (February 17, 2020-March 10, 2020); and (6) a focus on preventing imported cases (March 11, 2020-April 15, 2020).

**
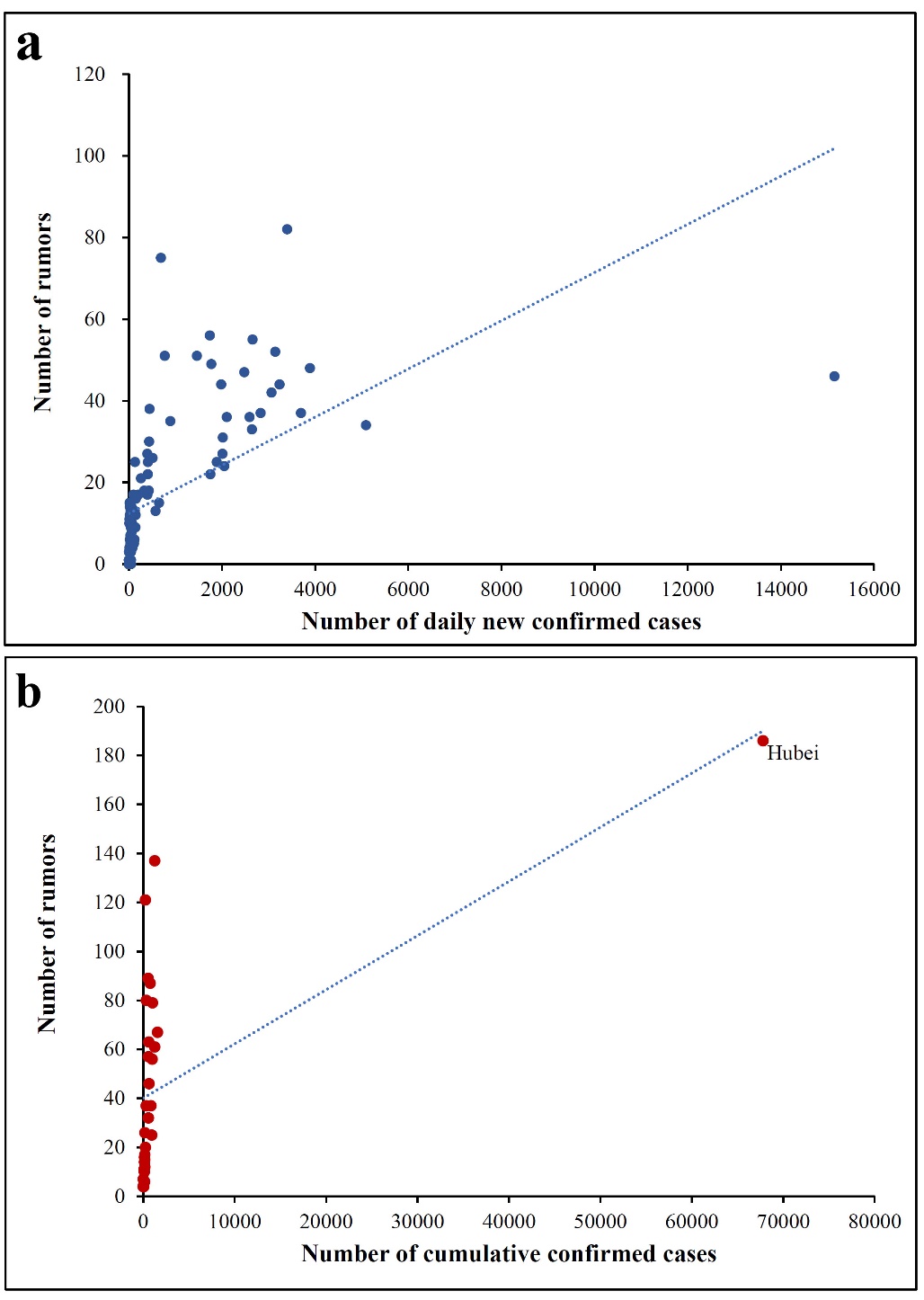
**

Figure S4. Relationship between number of rumors and the confirmed COVID-19 cases in China (a. daily new confirmed cases at national level from 12/30/2019 to 4/15/2020; b. cumulative confirmed cases at provincial level by 4/15/2020).

**
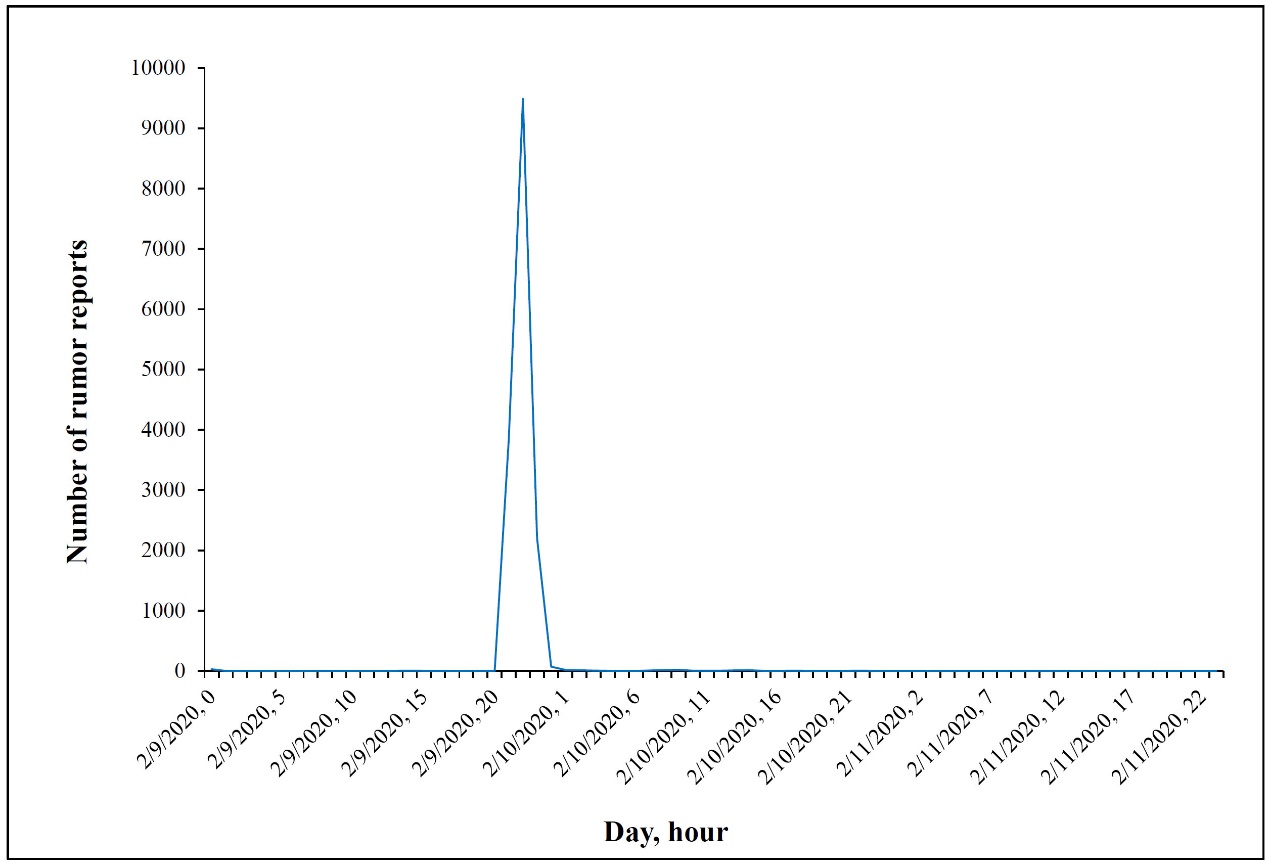
**

Figure S5. Number of rumor reports related to “*Case* *B*. the novel coronavirus is the SARS coronavirus” in hours.
